# Control of SARS-CoV-2 infection in rituximab-treated neuroimmunological patients

**DOI:** 10.1007/s00415-020-10046-8

**Published:** 2020-07-11

**Authors:** Marcel S. Woo, David Steins, Vivien Häußler, Matin Kohsar, Friedrich Haag, Birte Elias-Hamp, Christoph Heesen, Marc Lütgehetmann, Julian Schulze zur Wiesch, Manuel A. Friese

**Affiliations:** 1 Institute of Neuroimmunology and Multiple Sclerosis (INIMS), Center for Molecular Neurobiology Hamburg (ZMNH), University Medical Centre Hamburg-Eppendorf, Hamburg, Germany; 2 Division of Infectious Diseases, I. Department of Medicine, University Medical Center Hamburg-Eppendorf, Hamburg, Germany; 3 Department of Neurology, University Medical Center Hamburg-Eppendorf, Hamburg, Germany; 4 Department of Immunology, University Medical Center Hamburg-Eppendorf, Hamburg, Germany; 5 Private Neurological Practice, Hamburg, Germany; 6 Institute of Microbiology, Virology and Hygiene, University Medical Center Hamburg-Eppendorf, Hamburg, Germany; 7 German Center for Infection Disease (DZIF), University Medical Center Hamburg-Eppendorf, Hamburg, Germany

Dear Sirs,

Individuals with autoimmune diseases, such as multiple sclerosis (MS) or neuromyelitis optica spectrum disorder (NMOSD), that require long-term immunosuppression are regarded as particularly vulnerable in the current COVID-19 pandemic [1]. However, few details about the effect of individual immunotherapies have been reported, which could instruct us about the immunological control of severe acute respiratory syndrome coronavirus 2 (SARS-CoV-2). Specific antibodies are detectable within 2–19 days [2] and have been extensively analyzed for diagnostic purposes [3] and vaccine development [4]. It is unclear whether a durable antibody response is required for recovery of COVID-19 or whether it might even contribute to the pathogenesis by perpetuating hyperinflammation as has been shown for the closely related middle-east-respiratory-syndrome (MERS) coronavirus [5].

Here, we report on two individuals with underlying neuroimmunological diseases who were under stable rituximab therapy—a B cell-depleting monoclonal antibody [6, 7]—when confirmed COVID-19 developed. Infection with SARS-CoV-2 was verified in both cases by PCR.

Patient 1 was a 44-year-old woman with a history of breast carcinoma, which was treated by breast-conserving surgery in 2010 and a relapsing–remitting MS (diagnosed 1999; EDSS 2.0) that has been treated with rituximab since 2013 (last infusion in January 2020). She was admitted with malaise, muscle ache, cough, fever and mild dyspnea, which first developed during a ski-trip in a high-risk area on March 14th, 2020 and she was tested positive ten days later. On the day of admission, she showed elevated inflammatory biomarkers (CRP 34 mg/L, interleukin-6 371.9 ng/L, ferritin 292.7 µg/L), cardiac biomarkers (proBNP 253 ng/L) and D-dimers (0.61 mg/L) but normal procalcitonin (< 0.02 µg/L) and negative blood cultures. Radiologic findings of bilateral infiltrations indicated atypical pneumonia. On the second day of admission SARS-CoV-2 RNA was only detectable in pharyngeal swabs in low concentrations close to detection limit (Ct 37.4). Immunologically, she had normal lymphocyte counts (1.12 billion/mL) but absent B cells (not detectable, Supplementary Table 1). Serologically, we could not detect antibodies against SARS-CoV-2 IgG. The patient was clinically and serologically stable and was discharged after four days of inpatient symptomatic treatment against fever into home quarantine. Four weeks later, she electively visited our outpatient clinic and her PCR from a nasopharyngeal swab was now negative for SARS-CoV-2 RNA. Clinically, she was completely asymptomatic, and we did not observe neurological deterioration. Serologically, she was still negative for antibodies against SARS-CoV-2 IgG (Fig. 1a). A control X-ray of the chest showed a strong regression of pre-diagnosed bilateral pneumonic infiltrates.

**Fig. 1:**
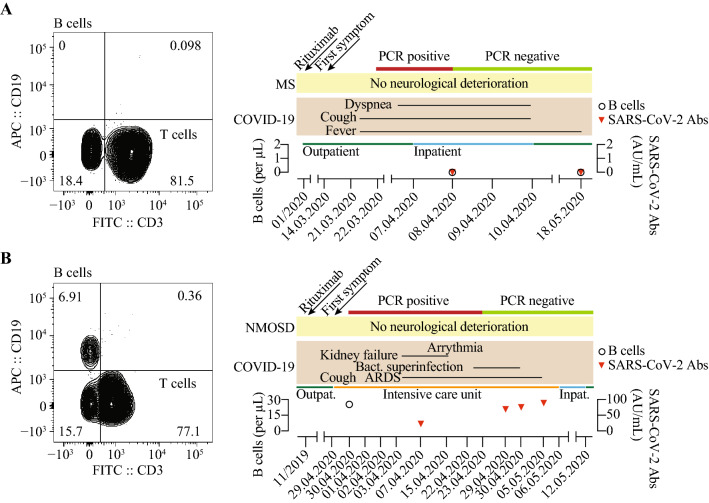
Summary of disease course, B cell count, PCR and antibody (Abs) response in patient 1 (**a**) and 2 (**b**)

Patient 2 was a 68-year-old female with neuromyelitis optica spectrum disorder (NMOSD, diagnosed 2014, EDSS 6.0), who was directly admitted to our intensive care unit (ICU) on March 29th, 2020 with progressive respiratory failure and infection of the urinary tract. She reported productive cough and anuria since the previous day. The patient was tested positive for SARS-CoV-2 by PCR on April 29th, 2020 (Ct 36). She had been receiving rituximab since 2014 and the last time in November 2020. Notably, the patient had well-treated hypothyroidism, myasthenia gravis in remission, well-adjusted insulin-dependent diabetes mellitus type 2, arterial hypertension, chronic obstructive pulmonary disease, obesity and has smoked daily 20 cigarettes for more than 15 years. On admission, inflammatory biomarkers (CRP 16 mg/L, interleukin-6 14.2 ng/L), cardiac parameters (CK 168 U/I, high sensitive troponin T 29 pg/mL, proBNP 546 ng/L) and d-dimers (2.93 mg/L) were elevated but procalcitonin (0.21 µg/L) was normal. Radiologic findings included bilateral pneumonic infiltrates and pleural effusions. She had a B cell count of 25/µL (Ref. 80–500/µL, Supplementary Table 2) at the day of admission and tested negative for SARS-CoV-2-specific antibodies (3.5 AU/mL; Ref. < 15 AU/mL) on April 7th, 2020, which converted to detectable antibodies on April 29th, 2020 (71.5 AU/mL). During her stay at our ICU she had a complicated disease course with bacterial superinfection and severe acute respiratory distress syndrome. She was intubated on April 1st, 2020 and subsequently received tracheotomy on April 17th, 2020 that was eventually removed on May 4th, 2020 after hemodynamic stabilization and decreasing infection parameters. Other complications included pre-renal failure due to volume depletion that was treated by intermittent continuous veno-venous hemodialysis and absolute tachyarrhythmia that was terminated by treatment with amiodaron. The patient completely recovered and was submitted to regular ward on May 6th, 2020. We did not observe a symptomatic exacerbation of her NMOSD and she was discharged on May 12th, 2020 (Fig. 1b).

In summary, we report on two patients who developed COVID-19 while under treatment with rituximab due to neuroimmunological diseases. Notably, their B cell count varied from non-detectable to markedly suppressed. We observed, that firstly only complete B cell depletion affected antibody response against SARS-CoV-2 and secondly, virologic control was possible in the absence of a detectable B cell response. Thirdly, neither of the two patients showed a clinical deterioration of their underlying neurological condition during or after SARS-CoV-2 infection. Thus, these two cases imply that immunological factors other than B cell-mediated antibody responses are required for COVID-19 control. However, for individuals with B cell depletion uncertainty remains towards the robustness of viral control, the degree of immunity and risk of reinfection.

## Electronic supplementary material

Below is the link to the electronic supplementary material.

Supplementary file1 (DOCX 36 kb)
