# Supplementary material for: Control of SARS-CoV-2 infection in rituximab-treated neuroimmunological patients
Source: J Neurol. 2020 Jul 11;268(1):5–7. doi: 10.1007/s00415-020-10046-8 (PMC7353821; doi:10.1007/s00415-020-10046-8)
Supplement: Supplementary file 1 — Supplementary file1 (DOCX 36 kb) [file 415_2020_10046_MOESM1_ESM.docx]

**Control of SARS-CoV-2 infection in rituximab-treated neuroimmunological patients**

**Marcel S. Woo^1,^*, David Steins, MD^2,^*, Vivien Häußler, MD^1,3^, Matin Kohsar^2^, Friedrich Haag, MD^4^, Birte Elias-Hamp, MD^5^, Christoph Heesen, MD^1,3^, Marc Lütgehetmann, MD^6^, Julian Schulze zur Wiesch, MD^2,7,^*, Manuel A. Friese, MD^1,^***

**Affiliations**

^1^Institute of Neuroimmunology and Multiple Sclerosis, University Medical Center Hamburg-Eppendorf, Germany

^2^Division of Infectious Diseases, I. Department of Medicine, University Medical Center Hamburg-Eppendorf, Germany

^3^Department of Neurology, University Medical Center Hamburg-Eppendorf, Germany

^4^Department of Immunology, University Medical Center Hamburg-Eppendorf, Germany

^5^Private neurology practice (BEH), Hamburg, Germany

^6^Institute of Microbiology, Virology and Hygiene, University Medical Center Hamburg-Eppendorf, Germany

^7^German Center for Infection Disease (DZIF), University Medical Center Hamburg-Eppendorf, Germany

*These authors contributed equally to the manuscript.

**Corresponding authors**

**Julian Schulze zur Wiesch, MD**

1. Department of Medicine, Infectious Diseases Unit

University Medical Center Hamburg Eppendorf

Martinistraße 52, 20246 Hamburg, Germany

Tel. +49(0)40 7410 52831

Email: j.schulze-zur-wiesch@uke.de

**Professor Manuel A. Friese, MD**

Institute of Neuroimmunology and Multiple Sclerosis (INIMS)

Center for Molecular Neurobiology Hamburg (ZMNH)

University Medical Centre Hamburg-Eppendorf

Falkenried 94, 20251 Hamburg, Germany

Tel. +49(0)40 7410 56615

Email: [manuel.friese@zmnh.uni-hamburg.de](mailto:manuel.friese@zmnh.uni-hamburg.de)

**Electronic Supplementary Material**

**Supplementary Table 1. Key laboratory data of patient 1.**

| Laboratory parameters (unit) | One day after admission  (April 8^th^, 2020) | Day of discharge  (April 10^th^, 2020) | Outpatient control (May 18^th^, 2020) |
| --- | --- | --- | --- |
| Vital parameters |  |  |  |
| - Oxygen saturation (%) | 98 | 94 | – |
| - Temperature (°C) | 36.8 | 38.1 | – |
| - Pulse rate | 66 | 63 | – |
| - Blood pressure | 110/60 | 100/60 | – |
| Hematology |  |  |  |
| - Hemoglobin (g/dL) | 13.3 | 13.8 | 11.8 |
| - Erythrocytes (billion/mL) | 4.64 | 4.8 | 4.2 |
| - Leucocytes (billion/mL) | 4.7 | 5.6 | 9.0 |
| - Thrombocytes (billion/mL) | 258 | 259 | 344 |
| - Neutrophils (%) | 67 | 61.3 | 63.5 |
| - Monocytes (%) | 6.9 | 7.4 | 7.7 |
| - Eosinophils (%) | < 0.1 | < 0.1 | 0.3 |
| - Basophiles (%) | < 0.1 | < 0.1 | 0.5 |
| Immunology |  |  |  |
| - Lymphocytes (total) (per µL) | 940 | – | 2337 |
| - Lymphocytes (relative) (%) | 23.7 | 28.6 | 26.9 |
| - T cells (total) (per µL) | 781 | – | 2113 |
| - T cells (relative) (%) | 83 | – | > 90 |
| - B cells (total) (per µL) | 0 | – | 0 |
| - B cells (relative) (%) | 0 | – | 0 |
| - CD4+ T cells (total) (per µL) | 398 | – | 587 |
| - CD4+ T cells (relative) (%) | 42 | – | 25 |
| - CD8+ T cells (total) (per µL) | 345 | – | 1475 |
| - CD8+ T cells (relative) (%) | 38 | – | 63 |
| - CD4 / CD8 ratio | 1.12 | – | 0.4 |
| Inflammatory parameters |  |  |  |
| - CRP (mg/L) | 33 | 29 | 26 |
| - Interleukin 6 (ng/L) | 371.9 | 287.2 | 414 |
| - Ferritin (µg/L) | 292.7 | 365.3 | 345.5 |
| - Pro-calcitonin (µg/L) | – | < 0.02 | < 0.02 |
| Coagulation parameters |  |  |  |
| - D-dimers (mg/L) | 0.61 | 0.74 | 0.2 |
| - INR (%) | 0.9 | – | 1.0 |
| - Fibrinogen (g/L) | > 3.7 | – | > 3.70 |
| - Antithombin III (%) | 93 | – | 106 |
| Cardiac parameters |  |  |  |
| - proBNP (ng/L) | 253 | 258 | 690 |
| - high-sensitivity troponin T (pg/mL) | – | – | – |
| - LDH (U/I) | 321 | 322 | 289 |
| - Creatine kinase (U/I) | 66 | – | 98 |
| Plasma |  |  |  |
| - Albumin (g/L) | 28.7 | – | 32.7 |
| - Bilirubin total (mg/dL) | 0.3 | – | 0.4 |
| - Creatinine (mg/dL) | 0.56 | 0.65 | 0.73 |
| - Urea (mg/dL) | 9.3 | 7.3 | 11.4 |
| - AST (GOT) (U/I) | 25 | 26 | 25 |
| - ALT (GPT) (U/I) | 19 | 21 | 15 |
| - GGT (U/I) | 17 | – | 19 |
| - Alkaline phosphatase (U/I) | 87 | – | 133 |

**Supplementary Table 2. Key laboratory data of patient 2.**

| Laboratory parameters (unit) | One day after admission  (March 30^th^) | One day before discharge  (May 11^th^) |
| --- | --- | --- |
| Vital parameters |  |  |
| - Oxygen saturation (%) | 85 | 96 |
| - Temperature (°C) | 39.1 | 36.2 |
| - Pulse rate | 66 | 64 |
| - Blood pressure | 124/69 | 140/70 |
| Hematology |  |  |
| - Hemoglobin (g/dL) | 9.6 | 8.7 |
| - Erythrocytes (billion/mL) | 3.59 | 3.09 |
| - Leucocytes (billion/mL) | 1.8 | 2.4 |
| - Thrombocytes (billion/mL) | 139 | 225 |
| - Neutrophils (%) | 58.1 | 55.1 |
| - Monocytes (%) | 9.1 | 8.1 |
| - Eosinophils (%) | 0.3 | 3.3 |
| - Basophiles (%) | 0.3 | 0.5 |
| Immunology |  |  |
| - Lymphocytes (total) (per µL) | 499 | – |
| - Lymphocytes (relative) (%) | 30.7 | 31.3 |
| - T cells (total) (per µL) | 411 | – |
| - T cells (relative) (%) | 82 | – |
| - B cells (total) (per µL) | 25 | – |
| - B cells (relative) (%) | 5 | – |
| - CD4+ T cells (total) (per µL) | 267 | – |
| - CD4+ T cells (relative) (%) | 53 | – |
| - CD8+ T cells (total) (per µL) | 131 | – |
| - CD8+ T cells (relative) (%) | 26 | – |
| - CD4 / CD8 ratio | 2.04 | – |
| Inflammatory parameters |  |  |
| - CRP (mg/L) | 12 | 24 |
| - Interleukin 6 (ng/L) | 10.7 | 5.4 |
| - Ferritin (µg/L) | 144 | 140.2 |
| - Pro-calcitonin (µg/L) | 0.21 | 0.04 |
| Coagulation parameters |  |  |
| - D-dimers (mg/L) | 2.93 | 0.94 |
| - INR (%) | 1 | – |
| - Fibrinogen (g/L) | 2.78 | – |
| - Antithombin III (%) | 94 | – |
| Cardiac parameters |  |  |
| - proBNP (ng/L) | – | 414 |
| - high-sensitivity troponin T (pg/mL) | 29 | 30 |
| - LDH (U/I) | 158 | 247 |
| - Creatine kinase (U/I) | 166 | – |
| Plasma |  |  |
| - Albumin (g/L) | 25.5 | – |
| - Bilirubin total (mg/dL) | < 0.2 | 0.2 |
| - Creatinine (mg/dL) | 5.54 | 1.04 |
| - Urea (mg/dL) | 68.3 | 12.7 |
| - AST (GOT) (U/I) | 28 | 24 |
| - ALT (GPT) (U/I) | 15 | 27 |
| - GGT (U/I) | 22 | – |
| - Alkaline phosphatase (U/I) | 28 | – |
